# Does Diabetes Increase the Frequency of Root-Filled teeth: Systematic Review and Meta-Analysis of Observational Studies

**DOI:** 10.4317/jced.61011

**Published:** 2023-11-01

**Authors:** María León-López, Daniel Cabanillas-Balsera, Jenifer Martín-González, Lucy J. Chandler-Gutiérrez, Victoria Areal-Quecuty, Paloma Montero-Miralles, Isabel Crespo-Gallardo, Juan J. Segura-Egea

**Affiliations:** 1 DDS, PhD. Department of Stomatology (Endodontic Section), School of Dentistry, University of Sevilla, C/ Avicena s/n, 41009-Sevilla, Spain; 2 DDS, MSc. Department of Stomatology (Endodontic Section), School of Dentistry, University of Sevilla, C/ Avicena s/n, 41009-Sevilla, Spain; 3 MD, DDS, PhD. Department of Stomatology (Endodontic Section), School of Dentistry, University of Sevilla, C/ Avicena s/n, 41009-Sevilla, Spain

## Abstract

**Background:**

The indicated treatment in cases of apical periodontitis (AP), a disease very prevalent in diabetic patients, is root canal treatment (RCT). This study aims to conduct a systematic review with meta-analysis to answer the following PICO question: In adult patients, does the absence or presence of diabetes affect the prevalence of root filled teeth (RFT)”?

**Material and Methods:**

PRISMA Guidelines have been followed to carry out this systematic review. A literature search was undertaken in PubMed-MEDLINE, Embase and Scielo. All studies reporting the prevalence of RFT in diabetic patients and control subjects using radiographic examination were included. Study characteristics and risk ratios with 95% CIs were extracted. Random-effects meta-analyses were performed.

**Results:**

Five studies fulfilled the inclusion criteria. Prevalence of RFT were estimated with 701 people and 15,882 teeth. Among diabetic patients, 6.1% of teeth had undergone RCT, while in controls this percentage was 3% (OR = 1.7; 95% CI = 1.0 – 2.9; p = 0.065). Among diabetic patients, 65% had at least one RFT, while in controls this percentage dropped to 55% (OR = 1.4; 95% CI = 0.5 – 3.7; *p*> 0.05). The certainty of evidence was low.

**Conclusions:**

The prevalence of RFT in diabetic patients is almost double that in the control population, however this result is only marginally significant. Dentists must take into account the high prevalence of RFT in diabetic patients, investigating the presence of diabetes in those patients in whom a high frequency of RCT is observed.

** Key words:**Diabetes, endodontics, epidemiology, root canal treatment, root filled teeth, prevalence, survey, population-based study.

## Introduction

When antigens and toxins from the necrotic and infected root canal invade the periapical tissue, an immune response is stimulated that manifests as periapical inflammation, termed apical periodontitis (AP) (1). The periapical inflammatory process will continue until the passage of antigens from the root canal ceases (2). The treatment indicated in cases of apical periodontitis is root canal treatment (RCT) (3). RCT removes the necrotic and infected contents of the root canal and seals the apical foramen. Thus, it stops the passage of antigens to periapical tissues and creates the conditions for the inflammatory tissue to become reparative tissue, with the consequent healing of the periapical lesion.

Since AP is a highly prevalent pathology throughout the world, affecting 5% of teeth, with at least one tooth affected by AP in 52% of the people (4), it would be expected that the prevalence of RCT would also be high. A recent systematic review with meta-analysis has found a prevalence of root filled teeth (RFT) of 8%, with 56% of people having at least 1 RFT (5).

On the other hand, there are numerous studies that have been published for more than two decades in which an association is found between endodontic pathology and various systemic diseases (6,7). Specially, diabetes is one of the systemic diseases on which more studies have investigated its possible association with AP (8-10). In diabetes, there is an alteration of metabolism that affects carbohydrates, lipids and proteins, its main sign being the increase in blood glucose, hyperglycemia (11). Increased blood glucose levels are associated with glucotoxicity, which is the main factor involved in the incidence and progression of serious microvascular complications associated with diabetes, such as diabetic retinopathy, nephropathy, and neuropathy (12).

It has been shown that the prevalence of AP in diabetic patients is higher than that of the general population (8,13). Therefore, it might be expected that the prevalence of RFT would also be high in these patients. Additionally, several systematic reviews have concluded that there is an association between diabetes and endodontic treatment outcome (14,15), with diabetes being considered an important preoperative prognostic factor for RCT, influencing negatively treatment outcome and RFT survival (7).

The aim of this study was to carry out a systematic review with meta-analysis to investigate the possible influence of diabetes on the frequency of RFT, including cross-sectional observational studies comparing a diabetic group and a healthy control group. The initial null hypothesis is that the frequency of RFT in diabetics is similar to that of the control subjects.

## Material and Methods

A protocol was prospectively preregistered at International prospective register of systematic reviews (PROSPERO) (CRD42023416903) (https://www.crd.york.ac.uk/prospero/export_details_pdf.php). The Preferred Reporting Items for Systematic Reviews and Meta-Analyses (PRISMA) guidelines have been followed to carry out this systematic review (16). The review focused on the following research question: Does the presence or absence of diabetes affect the prevalence of RFT in adult patients? PICOS (Population, Intervention, Comparison, Outcome, and type of study) schema for all the included studies to elaborate upon this research question were used to establish the eligibility criteria as follows:

Population: Adults patients.

Intervention: Presence of diabetes, diabetic.

Comparison: Absence of diabetes, healthy control subject.

Outcome: Prevalence of RFT.

Type of study: observational studies.

The main outcome was the percentage of RFT. As a secondary outcome it was taken into account the percentage of patients with at least one RFT.

-Data Sources and Searches

Once the PICO question and the eligibility criteria were established, the search strategy was designed. A literature search was undertaken without limits on time or language until 24th March 2023 in PubMed-MEDLINE, Embase and Scielo. Most cited descriptors in the previous publication on this theme were used in the electronic search strategy, using combining Medical Subject Heading (MeSH) terms and text word (tw). The search strategy can be found in the supplemental material.

A complementary screening was performed looking for any additional study on the references of the included studies that did not appear in the database search. Grey literature was searched but did not provide useful data (https://opengrey.eu/; https://scholar.google.com/; https://www.greynet.org/).

-Study Selection

The inclusion criteria established were (a) epidemiological studies published until 12th January 2023; (b) studies comparing diabetic patients with control healthy subjects; (c) studies reporting the prevalence of RFT in diabetic patients and control healthy subjects by radiographic examination (panoramic, periapical radiographs or cone beam computed tomography).

The following exclusion criteria were applied: (a) studies carried out in animals or in cell culture; (b) studies reporting data only from diabetic patients. (c) studies that did not report information about the prevalence of RFT.

Three authors (M.L.-L., D.C.-B., & J.J.S.-E.) selected the studies individually by screening the titles and abstracts. When the title and abstract did not allow judging the study, the full text was accessed. A second stage consisted of reading the full texts and judging the potential studies to be included based on the eligibility criteria. Disagreements on study inclusion were solved by consensus between the three authors. Duplicated studies in the databases search were considered only once.

-Data Extraction and Quality Assesment

The methodology of selected studies was examined, and main features were extracted and compiled including, authors, date of publication, study design, subjects and sample size, type of radiography used, main quantitative results, and odds ratio values.

The same three authors performed data extraction. The information related to publication were extracted: article’s identification (authors and year of publication); participants (gender, range and/or mean age of the sample and sample size); methods of image acquisition; results (number of teeth, number of RFT, number of people with at least one RFT, and distribution of RFT in the sample.

The quality of evidence of the included studies was analysed according to the guidelines provided by the Centre for Evidence-Based Medicine at Oxford http://www.cebm.net/index.aspx?o=5653. The risk of bias was assessed using the Newcastle-Ottawa Scale, adapted for cross-sectional studies (5).

Each of the included studies was evaluated for methodological risk of bias independently by four authors (M.L.-L., D.C.-B., V.A.-Q., & J.M.-G.). In case of disagreement, the authors discussed until they reached an agreement.

Two domains were taken into account when analysing the quality assessment and risk of bias of the individual studies: sample selection and outcome. The domain sample selection included the following items: representativeness of the sample, sample size, and non-respondents. The domain outcome included the following items: assessment of the outcome, inclusion of third molar in the outcome, inclusion of edentulous in total sample, and number of observers. The evaluation of each item was made according to the criteria previously described (5). Studies could score a maximum of 12 points; they were defined as high risk of bias if they scored 0 – 4 points, moderate risk of bias if they scored 5 – 8 points and low risk of bias if they scored 9 – 12 points.

Only dentate patients were taking into account for statistical analysis in studies that included edentulous patients in the sample.

-Data Synthesis and Analysis

The main outcome variable was the prevalence of RFT, calculated as the total number of RFT divided by the total number of teeth, expressed as a percentage. As a secondary outcome variable, the prevalence of diabetic patients with at least one RFT, expressed as a percentage, was also calculated. Odds ratio (OR), with its 95% confidence interval (CI) was calculated in every selected study trying to measure the effect of the relationship between diabetes and the prevalence of RFT. To determine the pooled OR and its 95% CI of the prevalence of RFT, the random-effect model meta-analysis was performed using the OpenMeta Analyst version 10.10 software (17), on the basis of inverse variance method. Another metanalysis was also performed using subgroup based on the number of diabetic patients with at least one RFT.

To estimate the variance and heterogeneity amongst trials, the Higgings I2 test were employed, considering a slight heterogeneity if it is between 25 and 50%, moderate between 50 and 75%, and high if >75% (18). Finally, a level of *p* = 0.05 was considered significant.

-Certainty of Evidence

The Grading of Recommendations Assessment, Development, and Evaluation (GRADE) tool was used to assess certainty of evidence (19). Two investigators (J.S.-E., D.C.-B.) independently carried out the assessment. High or moderate certainty of evidence can be interpreted as follows: it is very likely or probable that the true effect lies close to the estimated finding, and a recommendation can be made. Low or very low certainty of evidence indicates that our confidence in the result is limited or very weak, respectively.

## Results

-Literature Search Results

**Figure 1:**
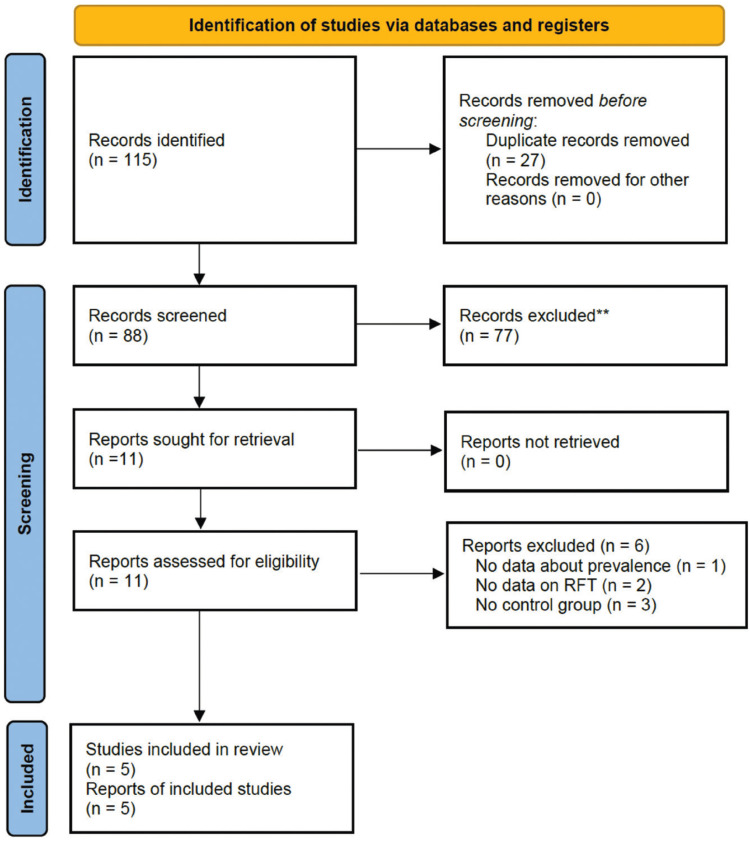
Flow diagram of the search strategy of the systematic review and metaanalysis following the Preferred Reporting Items for Systematic Reviews and Metaanalyses (PRISMA) guidelines 2020.

The flow diagram of literature search strategy and selected studies for this review is shown in Figure 1, according to PRISMA 2020 instructions. Initial search of different databases resulted in 115 published studies. After removal of duplicate studies (n = 27), 88 remained. After analyzing the titles and abstracts, 77 that did not investigate RFT were excluded. Ultimately, only 11 studies remained to read the full text.

After a thorough reading, one study that did not provide data on the prevalence of RFT was excluded (20). Two other studies (21,22) were also excluded because they did not refer to RCT. Three other studies were excluded because they only provided data on diabetic patients, without control group (9,23,24). Finally, five studies were selected for the systematic review and meta-analysis (8,13,25-27).

-Study Characteristics

The main characteristics of the five included studies (8,13,25-27) are summarized in Table 1. All studies were cross-sectional and level 4 evidence according to the Center for Evidence-Based Medicine at Oxford. Four of the studies included in their sample only type 2 diabetic patients (8,13,25,26) and the other study only included type 1 diabetic patients (27). In all studies the main outcome was to compare the prevalence of RFT in diabetic patients with that in healthy control subjects. Four of them also provided data on the percentage of patients with at least one RFT, both in diabetics and control subjects (8,13,25,27). Three studies used panoramic radiographs (8,26,27), one study used periapical radiographs (13), and the fifth both panoramic and periapical radiographs (25). In four of the studies diabetic patients had a lower mean number of teeth than the control group (8,13,25,27).

**Table 1:**
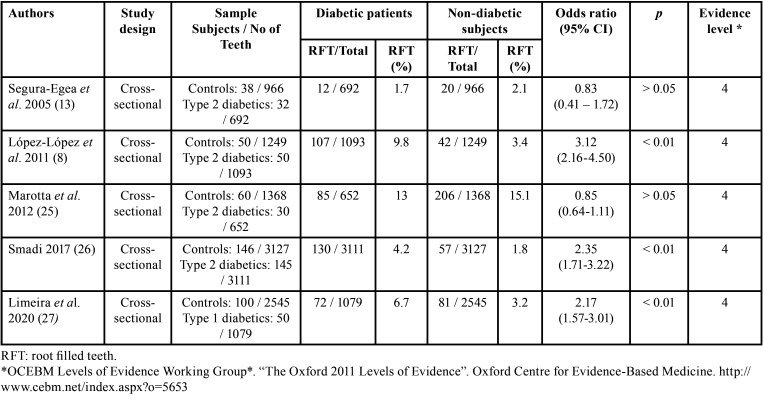
Descriptive characteristics of the included studies and extracted data.

Primary meta-analysis: Prevalence of RCT and diabetes 

Data from selected articles were analyzed and summarized in an evidence Table containing the data, descriptive statistics, and ORs calculated (Table 2). The five studies added a total of 701 people, who had 15,882 teeth, of which 812 (5.1%) were RFT (8,13,25-27).

**Table 2:**
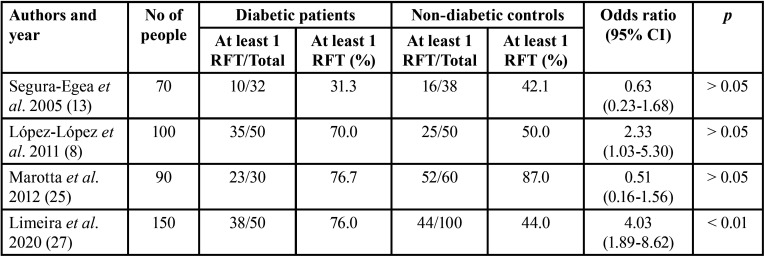
Percentage of people with at least one RFT in diabetic patients and control subjects in the four included studies.

**Figure 2:**
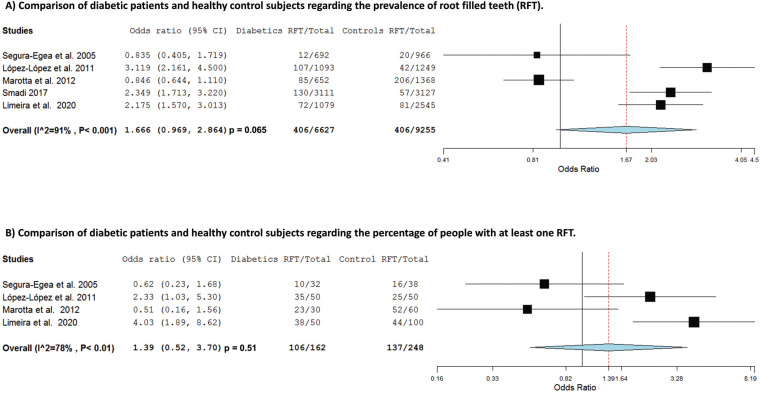
A) Forest plot of ORs and 95% confidence limits (CLs) for the comparison of diabetic patients and healthy control subjects regarding the prevalence of root filled teeth (RFT). Overall estimate is based on data from the five studies. Black squares represent the point estimates of the OR and have areas proportional to study size. Lines represent 95% confidence intervals. The diamond shows the summary statistics for the five studies. The solid line indicates an OR of 1.0, and the dashed line indicates the overall odds ratio. B) Forest plot of the studies that have calculated the percentage of people with at least one RFT in diabetic patients and control subjects.

Figure 2A shows the forest plot of the primary meta-analysis. Among diabetic patients, 6.1% of teeth had undergone RCT, while in healthy non-diabetic controls this percentage was 3.2%. The overall OR was calculated using DerSimonian–Laird method with random effects, resulting in an OR = 1.67 (95% CI = 0.97 – 2.86; *p* = 0.065), indicating that diabetic patients present a higher prevalence of RFT compared to control subjects. Heterogeneity value was I2 = 91%.

-Subgroup analysis: at least one RFT

A subgroup analysis was made including four studies (8,13,25,27) that provided information about patients with at least one RFT. The data can be found in the in the supplemental material. This meta-analysis included a total of 410 subjects, of which 162 were diabetic patients who had at least one RFT (Fig. 2B). Among diabetic patients, 65.4% had at least one RFT, while in healthy controls this percentage dropped to 55.2%. The OR calculated was 1.39 (95% CI = 0.52 – 3.71; *p* > 0.05). The heterogeneity value was I2 = 78%.

-Quality assessment and risk of bias

**Figure 3:**
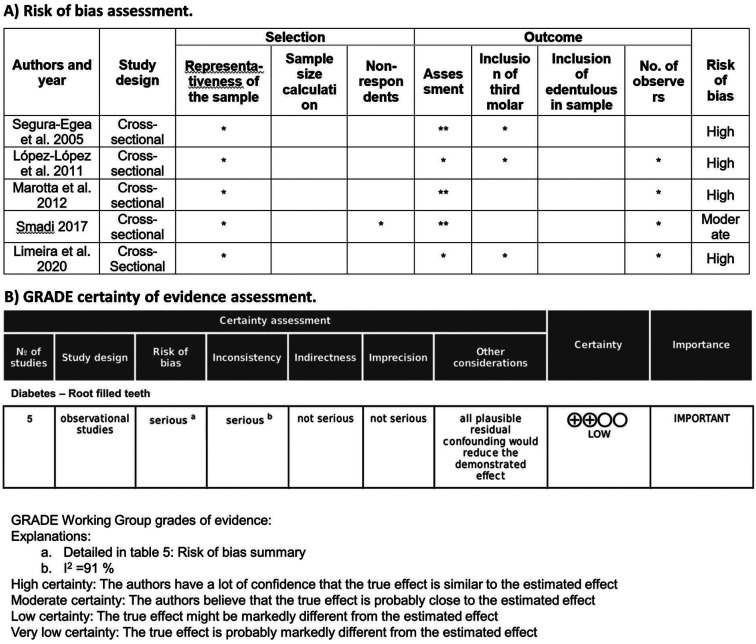
A) Quality assessment and risk of bias of individual studies assessed using the Newcastle-Ottawa Scale, adapted for cross-sectional studies. B) Certainty of evidence assessment by GRADE.

Quality assessment and risk of bias was evaluated for each study (Fig. 3A). Four of the five studies were classified as high risk of bias (8,13,25,27) and one of them was classified as moderate risk of bias (26). The certainty of evidence was rated as low (Fig. 3B).

## Discussion

This study aimed to conduct a systematic review and meta-analysis to investigate the possible association between diabetes and the frequency of RFT. The initial null hypothesis, i.e. that the prevalence of RFT in diabetics is similar to that of the general healthy population, must be accepted. It can be concluded that RFT are not associated to diabetes. Even though diabetic patients show almost twice (6.1%) as many RFT as control subjects (3.2%), with a calculated OR = 1.67, the *p* value is 0.065, not significant, although it could also be considered marginally significant. The percentage of diabetics with at least one RFT (65.4%) is also higher than that of healthy control subjects (55.2%) (OR = 1.39; *p* > 0.05), but not significant.

Taking into account the higher prevalence of AP that has been shown in diabetic patients (9,13,23), an increase in the frequency of RCT could be expected in the adult diabetic population. Moreover, diabetic patients have a high prevalence of periodontal disease, which is also associated with a higher prevalence of endodontic pathology tributary to RCT. Indeed, the high prevalence of RFT in diabetics observed in this study could even be underestimated, since there are numerous studies that show a higher prevalence of radiolucent periapical lesions (14) and loss of RFT in diabetic patients (15), compared to the healthy control population. This possible underestimation is also supported by the fact that in four (8,13,25,27) of the five studies included in the review the mean number of teeth is lower in diabetics than in control subjects.

It is quite possible that some of the teeth lost by diabetics were RFT. The persistence of AP in diabetics after RCT, possibly consecutive to a delay in the healing of periapical tissues, can lead frequently to tooth extraction. An umbrella review has concluded that diabetes is a risk factor for the outcome of RCT, therefore diabetes can be considered a key preoperative prognostic factor in endodontic treatment (28).

Additionally, diabetes and periodontal disease are closely linked and amplify one another, if not successfully controlled. Considering that periodontal disease is also a leading cause of tooth loss, the high prevalence of RFT in diabetics found in the present study, surely does not represent the true frequency of RCT in diabetics. Moreover, diabetic patients have increased periodontal disease in RFT and have a reduced likelihood of success of RCT. The combined effect of diabetes itself and periodontal disease may mask the real prevalence of RCT in diabetics, which is surely higher than that calculated in this study.

Regarding the articles included in the systematic review, the initial databases search provided 115 articles. When applying the inclusion criteria, resulted in a systematic review of five studies, all cross-sectional studies investigating the prevalence of RFT both in diabetic patients and in control subjects. Three of these studies (8,26,27) used panoramic radiographs to detect RFT, another (13) used periapical radiographs, and another one (25) used both periapical and panoramic radiographs. Although the detection of RFT can be performed with either of the two radiographic techniques, previous studies have found a higher prevalence of RFT with periapical radiographs (5).

A previous systematic review analysed the percentage of diabetic with RFT and the prevalence of RCT among diabetic patients around the world (29), concluding that 40% of diabetics have at least one RFT and more than 5.5% of teeth in diabetic patients were RFT. However, this study included only studies providing data on the frequency of RCT among diabetic patients, without comparing with control subjects. Therefore, it does not allow knowing if RCT is more frequent in diabetics than in the general population. On the contrary, the results of the present study, which reported 6.1% of RFT in diabetics and 65.4% of diabetics with at least 1 RFT, have been obtained with a different methodology: all the included studies compared the frequency of RFT in diabetic patients and control healthy subject. Therefore, the present study provides an odds ratio (OR) to assess the strength of the association between diabetes and RFT, thus being able to answer the question of whether RCT is more frequent in diabetics than in the general control population.

The results of this systematic review need to be carefully evaluated because its limitations. The high heterogeneity of the included studies (91% in the primary metaanalysis and 78% in the sub-group) indicate that studies differ substantially within study sampling and measurement variability. However, the pooled ORs were calculated using the random-effect model to estimate not a single correct overall answer to the research question but a distribution of particularized, situationally correct answers from an imagined universe of individual studies that might have been performed.

On the other hand, the quality of the studies included in a systematic review determines the reliability of the conclusions. The five articles included in the review are cross-sectional studies, whose level of evidence is four according to the Oxford classification. Regarding the risk of bias, four of the included studies were classified as high risk of bias (8,13,25,27), and another one was classified as moderate risk of bias (26). None of the included studies calculated the sample size, which is necessary to ensure a correct sample size to justify the study results. Moreover, most of the studies did not mention if edentulous patients were included in the sample, which alters the results of meta-analysis. Given the very low proportion of RCT performed on third molars, whether or not the third molar was included in the study does not represent a major limitation. So, low risk of bias was considered if the third molar was not included in the total patient sample. Similarly, if edentulous patients were not included in the total patient sample, low risk of bias was also considered. Nevertheless, when the study did not specify whether it included edentulous patients in the total sample, it was considered a very high risk of bias.

Another limitation of the present study is the low rate of included studies and patients. The reason lies in the fact that few studies follow a strict protocol for the selection of the individuals included in the sample. This is also the reason why none of the studies included in this systematic review and meta-analysis are at low risk of bias. All the listed limitations converge for the certainty of the evidence to have been rated as low, indicating that the true effect might be markedly different from the estimated effect (19).

The results of this systematic review with meta-analysis are of obvious clinical interest, and should be translated to the clinical practice. The verification of a high prevalence of RFT in diabetics is undoubtedly correlated with the higher incidence of AP found in diabetic patients (10), that leads them to request RCT more frequently than general population. Another reason that could justify a high prevalence of RFT in diabetics could be the fact that pulp pathology is not diagnosed early and is not treated in its initial phases, so that pulp infection progresses and ends up triggering AP and requiring RCT. A recent cross-sectional study has examined the prevalence of pulpal diagnoses in diabetic patients compared with nondiabetic control subjects (30). This study found higher prevalence of symptomatic irreversible pulpitis in young diabetic patients compared with control subjects, whereas in diabetics older than 60 years old, pulp necrosis was the predominant pulp diagnose. The reduction in the prevalence of symptomatic irreversible pulpitis and the increase in the prevalence of pulp necrosis with age could be explained with the senescent transformation of pulpal nerves. Then, the clinician must take in mind when caring for a diabetic patient, especially in older patients, that a careful clinical examination should be carried out to diagnose pulp pathology as early as possible to be able to establish the appropriate conservative treatment. Moreover, the endodontist should even investigate the presence of diabetes in those patients in whom a high frequency of RCT is observed.

Since diabetic patients have poor periapical healing and a tendency to non-retention of RFT (15), the prognosis of RCT may be poor. Therefore, dentists should suspect that the patient is an undiagnosed diabetic when multiple RCT failures are observed in the same patient. In addition, given this suspicion, a blood test should be requested to detect possible hyperglycaemia, and the additional complementary tests necessary to rule out the presence of diabetes.

## Conclusions

The systematic review and meta-analysis has shown an OR greater than 1.5, but not significant, in the association of diabetes with RFT in adult patients. This result are in correlation with the higher frequency of AP in the diabetic population. Endodontists must take into account the high prevalence of RFT in diabetic patients and follow up appropriately, investigating the presence of diabetes in those patients in whom a high frequency of RCT is observed.
